# Usefulness of computed tomography-measured psoas muscle thickness per height for predicting mortality in patients undergoing hemodialysis

**DOI:** 10.1038/s41598-021-98613-5

**Published:** 2021-09-24

**Authors:** Takahiro Yajima, Maiko Arao, Kumiko Yajima, Hiroshi Takahashi

**Affiliations:** 1 Department of Nephrology, Matsunami General Hospital, Gifu, 501-6062 Japan; 2 Department of Internal Medicine, Matsunami General Hospital, Gifu, 501-6062 Japan; 3 Division of Medical Statistics, Fujita Health University School of Medicine, Aichi, 470-1192 Japan

**Keywords:** Medical research, Nephrology

## Abstract

Computed tomography (CT)-measured psoas muscle thickness standardized for height (PMTH) has emerged as a promising predictor of mortality. The study aimed to investigate whether PMTH could accurately predict mortality in patients undergoing hemodialysis. We examined 207 patients (mean age: 63.1 years; men: 66.2%) undergoing hemodialysis for more than 6 months in hospital affiliated clinic. PMTH was calculated at the L3 vertebra level using CT. Patients were divided according to the PMTH cut-off points: 8.44 mm/m in women and 8.85 mm/m in men; thereafter, they were combined into low and high PMTH groups. PMTH was independently correlated with the simplified creatinine index (β = 0.213, *P* = 0.021) and geriatric nutritional risk index (β = 0.295, *P* < 0.0001) in multivariate regression analysis. During a median follow-up of 3.7 (1.8–6.4) years, 76 patients died, including 41 from cardiovascular causes. In the multivariate Cox regression analysis, low PMTH (adjusted hazard ratio, 2.48; 95% confidence interval, 1.36–4.70) was independently associated with an increased risk of all-cause mortality. The addition of binary PMTH groups to the baseline risk model tended to improve net reclassification improvement (0.460, p = 0.060). In conclusion, PMTH may be an indicator of protein energy wasting and a useful tool for predicting mortality in patients undergoing hemodialysis.

## Introduction

Muscle wasting, i.e., the loss of muscle mass, is prevalent in patients with end-stage kidney disease undergoing hemodialysis^1^. Muscle wasting is a result of a negative protein balance caused by inflammation, increased protein catabolism, and insufficient energy and protein intake^2^. Thus, muscle wasting may be a common component of protein energy wasting^3^, which is defined as a loss of muscle and fat mass due to chronic inflammation, or sarcopenia^4^, which is characterized by low skeletal muscle mass with low muscle function. Both protein energy wasting and sarcopenia are prevalent and associated with increased mortality in patients undergoing hemodialysis^3, 5–7^. Because muscle function, muscle strength, and physical performance are generally low in patients undergoing hemodialysis, the precise measurement of muscle mass is clinically important^8^. Surrogate methods such as bioelectrical impedance analysis and dual-energy X-ray absorptiometry are available in clinical practice; however, the accuracy of these methods can be affected by the patient’s hydration status^9, 10^. Thus, the European Consensus Statement recommends computed tomography (CT) as the gold standard method for the assessment of muscle mass, as it is not affected by hydration status^4, 11^.

Several previous studies have reported that CT-measured indices such as the abdominal skeletal muscle index and psoas muscle mass index at the level of the third lumbar vertebra (L3) are widely used to diagnose sarcopenia or muscle wasting and predict mortality in patients with various cancers and chronic liver disease^12–14^. However, specialized software, multiple attempts, and specific technical skills are required to measure these indices. Recently, CT-measured psoas muscle thickness per height (PMTH), defined as the largest transverse diameter of the right psoas muscle standardized for height, has been introduced as a simple measurable indicator of skeletal muscle mass for predicting mortality in patients with advanced liver disease^15–19^. However, in the field of nephrology, the use of CT for body composition analysis is limited owing to radiation exposure and high costs.

Only a few studies have examined the association between CT-measured sarcopenia indices and mortality in patients undergoing hemodialysis^20–22^. Although the use of PMTH for predicting mortality remains unclear, we hypothesized that low PMTH may be an increased risk of mortality in this population. It is clinically important to consider the pathophysiology which brings to lowering PMTH, therefore the relationships between PMTH and baseline variables were examined. Moreover, the present study aimed to investigate whether CT-measured PMTH can accurately predict all-cause and cardiovascular mortality in patients undergoing maintenance hemodialysis.

## Results

### Baseline characteristics

The baseline characteristics of the study participants are summarized in Table 1. The average age was 63.1 ± 13.6 years, and 66.2% were men. The median hemodialysis vintage was 2.1 (0.9–5.1) years. History of cardiovascular disease was present in 64.3% of the participants. The geriatric nutritional risk index (GNRI), simplified creatinine index (SCI), and C-reactive protein (CRP) concentrations were 93.8 ± 8.1, 20.6 ± 3.1 mg/kg/day, and 0.16 (0.07–0.53) mg/dL, respectively. The mean psoas muscle thickness (PMT) and PMTH were 15.5 ± 6.4 mm and 9.6 ± 3.8 mm/m, respectively. The PMT and PMTH were significantly higher in men than in women (17.3 ± 6.4 mm vs. 12.0 ± 4.7 mm and 10.5 ± 3.8 mm/m vs. 7.8 ± 3.0 mm/m, respectively; both *P* < 0.0001).

**Table 1:** Baseline characteristics of the study participants.

	All patients (*N* = 207)	Low PMTH group (*N* = 95)	High PMTH group (*N* = 112)	*P-*value
Age (years)	63.1 ± 13.6	67.8 ± 11.8	59.1 ± 13.8	< 0.0001
Men (%)	66.2	55.8	75.0	< 0.0001
**Underlying kidney disease**				0.46
Diabetic kidney disease (%)	42.5	36.8	47.3	
Chronic glomerulonephritis (%)	30.9	33.7	28.6	
Nephrosclerosis (%)	19.8	20.0	19.6	
Others (%)	6.8	9.5	4.5	
Hemodialysis vintage (years)	2.1 (0.9–5.1)	2.1 (0.8–5.6)	2.0 (1.0–5.0)	0.89
Alcohol (%)	20.3	17.9	22.3	0.13
Smoking (%)	23.7	16.8	29.5	0.0028
Hypertension (%)	95.2	91.6	98.2	0.027
Diabetes (%)	45.4	41.1	49.1	0.23
History of CVD (%)	64.3	69.5	59.8	0.45
Dry weight (kg)	57.2 ± 12.8	51.9 ± 10.6	61.7 ± 12.8	< 0.0001
Height (cm)	160.5 ± 8.7	158.2 ± 8.3	162.5 ± 8.7	< 0.0001
Body mass index (kg/m^2^)	22.1 ± 4.0	20.6 ± 3.4	23.3 ± 4.2	< 0.0001
Blood urea nitrogen (mg/dL)	57.9 ± 14.6	55.6 ± 16.3	59.9 ± 12.9	0.023
Creatinine (mg/dL)	9.5 ± 3.1	8.4 ± 2.8	10.4 ± 3.0	< 0.0001
Albumin (g/dL)	3.7 ± 0.5	3.5 ± 0.5	3.8 ± 0.3	< 0.0001
Hemoglobin (g/dL)	10.5 ± 1.4	10.4 ± 1.5	10.7 ± 1.4	0.16
Total cholesterol (mg/dL)	150 ± 33	148 ± 34	152 ± 33	0.47
Uric acid (mg/dL)	6.9 ± 1.7	6.8 ± 1.9	7.1 ± 1.5	0.19
Calcium (mg/dL)	9.0 ± 0.8	8.9 ± 0.9	9.0 ± 0.8	0.27
Phosphorus (mg/dL)	5.0 ± 1.4	4.7 ± 1.4	5.2 ± 1.4	0.020
iPTH (pg/mL)	113 (48–187)	98 (35–154)	130 (49–217)	0.16
Glucose (mg/dL)	144 ± 60	141 ± 57	147 ± 63	0.40
CRP (mg/dL)	0.16 (0.07–0.53)	0.30 (0.10–1.25)	0.13 (0.06–0.26)	0.0004
Kt/V urea	1.4 ± 0.3	1.4 ± 0.3	1.3 ± 0.3	0.0054
SCI (mg/kg/day)	20.6 ± 3.1	19.3 ± 2.7	21.7 ± 3.0	< 0.0001
GNRI	93.8 ± 8.1	90.1 ± 8.8	96.5 ± 6.1	< 0.0001
PMT (mm)	15.5 ± 6.4	10.4 ± 2.6	19.9 ± 5.3	< 0.0001
PMTH (mm/m)	9.6 ± 3.8	6.5 ± 1.5	12.2 ± 3.1	< 0.0001

*PMTH* psoas muscle thickness per height, *CVD* cardiovascular disease, *iPTH* intact parathyroid hormone, *CRP* C-reactive protein, *SCI* simplified creatinine index, *GNRI* geriatric nutritional risk index, *PMT* psoas muscle thickness.

### Agreements for PMT measurements

The intra- and inter-operator reproducibility of CT-measured PMT were assessed in a randomly selected sample of 30 patients; the measurements were performed in a blinded manner by two investigators (TY and MA). The intra-observer ICCs for the PMT measurements were 0.99 (95% confidence interval [CI] 0.99–0.99) for TY and 0.99 (95% CI 0.99–0.99) for MA. The interobserver intraclass correlation coefficient (ICC) for the PMT measurements was 0.98 (95% CI 0.95–0.99).

### Associations between PMTH and baseline variables

In the univariate linear regression analysis, the PMTH was significantly and negatively associated with age and log CRP. Conversely, the PMTH was significantly and positively associated with sex (male), GNRI, and SCI. The correlation coefficients among all explanatory variables were less than 0.66. The VIFs were less than 2.56. Multivariate regression analysis revealed that PMTH was independently associated with sex (male; β = 0.202, *P* = 0.0025), SCI (β = 0.213, *P* = 0.021), and GNRI (β = 0.295, *P* < 0.0001) (Table 2).

**Table 2:** Regression analyses of the associations between psoas muscle thickness per height and baseline variables.

Variables	Univariate analysis		Multivariate analysis	
	r	*P*-value	β	*P*-value
Age	− 0.359	< 0.0001	− 0.093	0.26
Male sex	0.335	< 0.0001	0.202	0.0025
Log CRP	− 0.221	0.0014	− 0.010	0.87
SCI	0.494	< 0.0001	0.213	0.021
GNRI	0.473	< 0.0001	0.295	< 0.0001

*CRP* C-reactive protein, *SCI* simplified creatinine index, *GNRI* geriatric nutritional risk index.

### Associations of PMTH with all-cause and cardiovascular mortality

**Figure 1:**
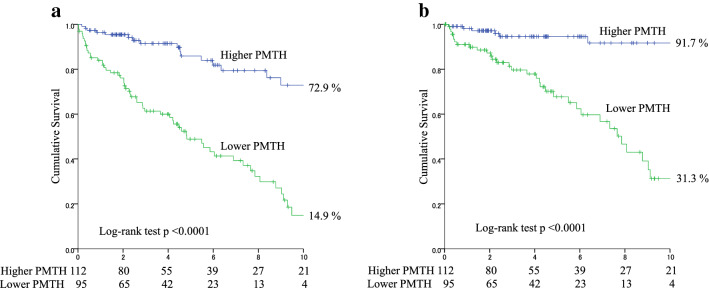
Kaplan–Meier survival curves. The all-cause mortality **(a)** and cardiovascular mortality **(b)** between the low PMTH and high PMTH groups were compared. *PMTH* psoas muscle thickness per height.

During the median follow-up period of 3.7 (1.8–6.4) years, 76 patients died [cardiovascular disease (CVD), n = 41 (53.9%); infection, n = 19 (25.0%); cancer, n = 4 (5.3%); and others, n = 12 (15.8%)]. In the univariate Cox proportional hazards analysis, the PMTH was a significant predictor of all-cause mortality [hazard ratio (HR), 0.78; 95% CI, 0.71–0.84; *P* < 0.0001]. Furthermore, the PMTH was a significant predictor of all-cause mortality in men (HR 0.72; 95% CI 0.64–0.80, *P* < 0.0001) and women (HR 0.82; 95% CI 0.69–0.95; *P* = 0.0073). To maximize the predictive value of PMTH for all-cause mortality in men and women, the receiver operating characteristic (ROC) analysis was performed and cut-off values of 8.44 mm/m in women (sensitivity, 0.489; specificity, 0.751; area under the curve 0.634; *P* = 0.042) and 8.85 mm/m in men (sensitivity, 0.837; specificity, 0.765; area under the curve, 0.846; *P* < 0.0001) were obtained. The 10-year survival rates were 72.9% for the high PMTH group and 14.9% for the low PMTH group (*P* < 0.0001) (Fig. 1). In multivariate Cox proportional hazards analysis adjusted for sex and age, history of CVD, SCI, GNRI, and log CRP, which were found to be significant factors in the univariate analysis, PMTH was an independent predictor of all-cause mortality (adjusted HR, 0.86; 95% CI, 0.79–0.94; *P* = 0.0014). There was no interaction between PMTH and sex for predicting mortality (p = 0.26). A low PMTH (adjusted HR, 2.48; 95% CI, 1.36–4.70; *P* = 0.0027) was independently associated with an increased risk of all-cause mortality (Table 3). Similar results were obtained for cardiovascular mortality (Table 3).

**Table 3:** Cox proportional hazards analysis of the psoas muscle thickness per height for prediction of all-cause and cardiovascular mortality.

Variables	Univariate analysis				Multivariate analysis ^a^			
	Regression coefficient	Baseline hazard	HR (95% CI)	*P*-value	Regression coefficient	Baseline hazard	HR (95% CI)	*P*-value
All-cause mortality								
PMTH (continuous)	−0.25	7.81	0.78 (0.71–0.84)	< 0.0001	−0.15	44.09	0.86 (0.79–0.94)	0.0014
Low PMTH	1.64	0.34	5.16 (3.01–8.82)	< 0.0001	0.91	15.08	2.48 (1.36–4.70)	0.0027
**Cardiovascular mortality**								
PMTH (continuous)	−0.29	5.72	0.75 (0.66–0.84)	< 0.0001	−0.21	7.29	0.81 (0.71–0.94)	0.0023
Low PMTH	2.04	0.14	7.69 (3.38–17.51)	< 0.0001	1.49	1.51	4.23 (1.77–11.47)	< 0.0001

*HR* hazard ratio, *CI* confidence interval, *PMTH* psoas muscle thickness per height.

^a^Adjusted for sex, age, history of cardiovascular disease, simplified creatinine index, geriatric nutritional risk index, and log C-reactive protein concentration, which were found to be significant in the univariate analysis.

### Model discrimination

When compared with an established risk model including sex, age, a history of CVD, SCI, GNRI, and log CRP, the addition of binary PMTH did not improve Harrell’s C-index for all-cause mortality (0.828 to 0.833, p = 0.76). However, the time dependent NRI and IDI tended to be improved (0.460, p = 0.060, and 0.064, p = 0.053), respectively (Table 4). Similar results were obtained for cardiovascular mortality (Table 4).

**Table 4:** Predictive accuracy of the psoas muscle thickness per height for all-cause and cardiovascular mortality using Harrell’s C-index for Cox hazard model, time dependent NRI and time dependent IDI.

Variables	Harrell’s C-index	*P*-value	Time dependent NRI	*P*-value	Time dependent IDI	*P*-value
**All-cause mortality**						
Established risk factors^a^	0.828		Reference		Reference	
+ PMTH (continuous)	0.834	0.79	0.396	0.080	0.056	0.053
+ PMTH (low vs. high)	0.833	0.76	0.460	0.060	0.064	0.053
**Cardiovascular mortality**						
Established risk factors^a^	0.845		Reference		Reference	
+ PMTH (continuous)	0.855	0.83	0.454	0.073	0.075	0.073
+ PMTH (low vs. high)	0.854	0.49	0.663	0.073	0.121	0.060

*NRI* net reclassification improvement, *IDI* integrated discrimination improvement, *PMTH* psoas muscle thickness per height.

^a^Sex, age, a history of cardiovascular disease, simplified creatinine index, geriatric nutritional risk index, and log C-reactive protein concentration.

## Discussion

The main findings of the present study were that CT-measured PMTH was independently associated with SCI and GNRI, which were markers of protein energy wasting, and that a low PMTH was independently associated with an increased risk of all-cause and cardiovascular mortality in patients undergoing hemodialysis. Therefore, PMTH may be an indicator of protein energy wasting and, consequently, a simple and useful tool for accurately predicting mortality in this population.

In patients undergoing hemodialysis, muscle wasting may be partially explained by protein energy wasting^2^, a malnourished state of negative protein balance, characterized by the loss of body protein (muscle) and fuel reserves (fat) due to catabolic inflammation, which is prevalent and associated with an increased risk of mortality in this population^3, 23^. We previously reported that high muscle mass and/or fat mass measured by bioelectrical impedance analysis are significantly associated with a decreased risk of all-cause mortality^28^. As a marker of protein energy wasting, GNRI, a simple objective nutritional assessment index that is calculated using only the serum albumin and actual/ideal body weight ratio, is widely used for patients undergoing hemodialysis^24–28^. The SCI, determined by age, sex, single pool Kt/V for urea, and pre-dialysis serum creatinine level, is used as a surrogate marker of muscle mass or as a valuable tool for observation of nutritional status in hemodialysis patients^29–31^. Tsai et al.^32^ recently reported that SCI was an independent predictor of the presence of protein energy wasting. Moreover, Yamada et al.^33^ demonstrated that GNRI and SCI equally predicted all-cause mortality in hemodialysis patients. In the present study, CT-measured PMTH was independently and positively associated with both SCI and GNRI, suggesting that PMTH may be an indicator of protein energy wasting.

Recently, CT-measured PMTH has emerged as a marker of muscle wasting as well as a prognostic indicator of mortality in patients with liver disease^15–19^. Compared with conventional CT-based sarcopenic indices measured at the L3 level, this novel method has advantages such as no requirement of specific software or technical skills. However, it also has some issues that need to be addressed before it is standardized for use. Durand et al.^15^ and Huguet et al.^16^ reported that CT-measured PMTH at the umbilicus level predicts mortality in patients with liver cirrhosis. Considering sex-related differences in muscle distribution and structure, Gu et al.^17^ and Praktiknjo et al.^18^ proposed sex-specific cut-off points for PMTH at the umbilicus level. They demonstrated that the use of sex-specific cut-off points improves the prognostic value of PMTH in patients with cirrhosis. However, the level of the umbilicus is heterogeneously located from L3 to L5. Thus, Paternostro et al.^19^ recently proposed a sex-specific standardized measurement of PMTH at the L3 level, where conventional CT-based sarcopenic indices are measured, and showed that PMTH is superior to conventional CT-measured sarcopenic indices in predicting mortality of patients with cirrhosis.

CT may be the gold standard for the assessment of muscle mass^4, 11^ even in patients undergoing hemodialysis because CT is not affected by the patient’s hydration status. Several studies in Japan evaluated the association between CT-measured sarcopenic indices and prognosis in patients undergoing hemodialysis. Fukasawa et al.^20^ reported that the dry weight–adjusted thigh muscle area, but not the abdominal muscle area, is a predictor of mortality. Kurumisawa et al.^21^ demonstrated that the CT-measured psoas muscle index, assessed before cardiovascular surgery, is a predictor of long-term survival after cardiac surgery. Similarly, Takata et al.^22^ reported that the CT-measured psoas muscle index is correlated with the bioelectrical impedance analysis–measured skeletal muscle mass index, where a low psoas muscle index is associated with an increased risk of mortality. Moreover, they suggested that the psoas muscle index assessed using CT can be an alternative to bioelectrical impedance analysis in patients undergoing hemodialysis. However, the relationship between CT-measured PMTH, a new CT-measured sarcopenic index, and mortality remains unknown. To our knowledge, our study is the first to analyze the relationship between CT-measured PMTH and mortality in patients undergoing hemodialysis. In the present study, a lower PMTH was independently associated with an increased risk of all-cause and cardiovascular mortality, even after adjusting for SCI and GNRI. In addition, the addition of PMTH on predicting model with established risk factors tended to improve the NRI and IDI, however, did not reach at statistical significance, probably due to small number of patients. These should be re-evaluated in larger population.

Our study has several limitations. First, the present retrospective, single-center study included a relatively small number of patients undergoing hemodialysis. Second, all enrolled patients were Japanese, who reportedly have a better prognosis that hemodialysis patients in the United States of America and Europe^34^. Thus, the results might not be generalizable to patients from other countries undergoing hemodialysis. Third, only PMTH data measured at enrolment were used for the analysis; therefore, any changes in these values during the long-term follow-up period were not considered. Further prospective, large-scale, multicenter studies are needed to validate our results.

In conclusion, CT-measured PMTH at the L3 level was independently associated with SCI and GNRI, and a low PMTH was independently associated with an increased risk of all-cause and cardiovascular mortality in patients undergoing hemodialysis. Therefore, PMTH may be an indicator of protein energy wasting and could be considered a simple and useful tool for accurately predicting mortality in this population.

## Methods

### Study participants

A total of 231 patients undergoing hemodialysis for longer than 6 months and who had undergone abdominal CT to detect early-stage renal cell carcinoma between January 2008 and December 2018 at the outpatient clinic of Matsunami General Hospital were screened. Twenty-four patients with a history of cancer were excluded; thus, 207 patients were ultimately enrolled in the present study. In our hospital, abdominal CT was performed as a screening test to detect early-stage renal cell carcinoma within 1 year after either the initiation of hemodialysis or transfer to our hospital. For this retrospective study, the requirement for informed consent was waived by the ethics committee of Matsunami General Hospital because patient data were anonymized. This study adhered to the principles of the Declaration of Helsinki, and the study protocol was approved by the ethics committee of Matsunami General Hospital (No. 484).

### Data collection

The following data were collected from the medical charts of the study participants: age; sex; underlying kidney disease; hemodialysis vintage; history of alcohol use, smoking, hypertension, diabetes, and CVD; dry weight; and height. In this study, CVD included angina pectoris, myocardial infarction, heart failure, stroke, and peripheral artery disease. Hypertension was defined as the use of antihypertensive drugs or a systolic blood pressure ≥ 140 mm Hg and/or a diastolic blood pressure ≥ 90 mm Hg prior to hemodialysis. Diabetes was defined as the use of glucose-lowering medication or a history of diabetes. Blood samples were obtained prior to the initiation of a hemodialysis session with the patient in the supine position, and the laboratory data that were extracted for data analysis were from the month when the CT was performed.

The GNRI was calculated from serum albumin level, dry weight, and height as follows: GNRI = 14.89 × serum albumin (g/dL) + 41.7 × (dry weight/ideal body weight)^25,35^. Ideal body weight (kg) was calculated as 22 × [height (m)]^2^. When dry weight was equal to or exceeded the ideal body weight, the ratio of dry weight to ideal body weight (dry weight/ideal body weight) was set to 1. The SCI was calculated from sex, age, single pool Kt/V for urea, and serum creatinine level as follows: SCI (mg/kg/day) = 16.21 + 1.12 × [0 for woman; 1 for man] – 0.06 × age (years) – 0.08 × single pool Kt/V for urea + 0.009 × serum creatinine (μmol/L)^29^.

### Measurement of PMTH

The PMT was determined using abdominal CT that was performed after a hemodialysis session using a wide-bore, 16-slice multidetector CT scanner (LightSpeed RT16; GE Healthcare, Waukesha, WI, USA), yielding 5-mm-thick slices. A cross-sectional CT image at the level of L3 was selected, and the transverse thickness of the right psoas muscle was measured using XTREK View (J-Mac System Inc., Sapporo, Japan). The transverse PMT was defined as the diameter of the psoas muscle perpendicular to the axial diameter, as previously reported^15–19^. The PMT was standardized for height to obtain the PMTH: PMTH (mm/m) = PMT (mm)/height (m).

### Follow-up study

The primary endpoint was all-cause mortality, and the secondary endpoint was cardiovascular mortality. Patients were followed up until December 2019. In this study, follow-up period was defined as the interval from the date when CT was performed to the date of death or the date when transferred to another hemodialysis unit. Patients who were alive were censored in December 2019.

### Statistical analysis

Normally distributed variables were expressed as means ± standard deviations, while non-normally distributed variables were expressed as medians and interquartile ranges. The Shapiro–Wilk test and visual inspection were used to examine whether a variable is normally distributed. ROC analysis was performed to assess the sensitivity and specificity of PMTH for predicting 10-year all-cause mortality in each sex. The optimal sex-specific cut-off values of PMTH were determined at the point to maximize Youden’s J statistic. Thereafter, patients were combined into low and high PMTH groups. The differences between the low and high PMTH groups were compared using Student’s *t*-test or the Wilcoxon rank sum test for continuous variables and the chi-squared test for categorical variables. Intra- and interobserver reproducibility for the measurement of PMT using CT were evaluated using the ICC and 95% CIs.

Univariate linear regression analysis was performed to examine the baseline factors associated with PMTH. Subsequently, multivariate regression analysis was performed using the factors found to be significant (*P* < 0.05) in the univariate analysis. As assumptions for multivariate linear regression analysis, continuous variables which were non-normally distributed were log-transformed. To confirm the multi-collinearity, correlation coefficients were calculated among all explanatory variables. Variance Inflation Factors (VIFs) were also calculated. The Kaplan–Meier method was used to estimate survival, and the difference of the survival was compared using the log-rank test. HRs and 95% CIs for all-cause and cardiovascular mortality were examined using Cox proportional hazard regression analysis. Since the distribution of the PMTH values differed according to sex, interaction between PMTH and sex was examined, and sex was included in the multivariate analysis.

To determine whether the prediction of all-cause and cardiovascular mortality improved after the addition of PMTH to the baseline model with sex and the covariates that were found to be significant in the univariate analysis, Harrell’s C-index for Cox regression model^36, 37^, time dependent net reclassification improvement (NRI)^38^ and time dependent integrated discrimination improvement (IDI)^39^ were calculated with likelihood-ratio tests using R version 4.04. The NRI is a relative indicator of the number of patients for whom the predicted probability of mortality improves, whereas the IDI represents the average improvement in predicted probabilities for mortality after the addition of variables to the baseline model. All statistical analyses were performed using SPSS version 21 (IBM Corp., Armonk, NY, USA) and R version 4.04. Statistical significance was set at *P* < 0.05.

### Ethics declarations

This study adhered to the principles of the Declaration of Helsinki, and the study protocol was approved by the ethics committee of Matsunami General Hospital (No. 484). The requirement for informed consent was waived because patient data were anonymized.

## Data Availability

The dataset analyzed in the present study is available from corresponding author on reasonable request.
